# The temporal dynamics and clinical relevance of choroid plexus measures in multiple sclerosis

**DOI:** 10.1093/braincomms/fcaf239

**Published:** 2025-06-14

**Authors:** Ermelinda De Meo, Sarmad Al-Araji, Baris Kanber, Alessia Bianchi, Charmaine Hiu-Ying Yam, Ronja Christensen, Suraya Mohamud, Jed Wingrove, Omar Abdel-Mannan, Anuriti Aojula, Dimitrios Champsas, Weaam Hamed, Ahmed Hammam, Dominic Wilkins, Anna He, Yael Hacohen, Ferran Prados, Frederik Barkhof, Declan Chard, Olga Ciccarelli

**Affiliations:** Queen Square MS Centre, Department of Neuroinflammation, UCL Queen Square Institute of Neurology, London, WC1B 5EH, UK; NEUROFARBA Department, University of Florence, 50100 Florence, Italy; Queen Square MS Centre, Department of Neuroinflammation, UCL Queen Square Institute of Neurology, London, WC1B 5EH, UK; National Institute for Health and Care Research (NIHR) University College London Hospitals (UCLH) Biomedical Research Centre, London, WC1E 6BT, UK; Centre for Medical Image Computing, Department of Medical Physics and Biomedical Engineering, University College London, London, WC1V 6LJ, UK; Queen Square MS Centre, Department of Neuroinflammation, UCL Queen Square Institute of Neurology, London, WC1B 5EH, UK; Queen Square MS Centre, Department of Neuroinflammation, UCL Queen Square Institute of Neurology, London, WC1B 5EH, UK; Neurology Department, Cleveland Clinic London, London, SW1X 7HY, UK; Queen Square MS Centre, Department of Neuroinflammation, UCL Queen Square Institute of Neurology, London, WC1B 5EH, UK; Queen Square MS Centre, Department of Neuroinflammation, UCL Queen Square Institute of Neurology, London, WC1B 5EH, UK; Queen Square MS Centre, Department of Neuroinflammation, UCL Queen Square Institute of Neurology, London, WC1B 5EH, UK; Queen Square MS Centre, Department of Neuroinflammation, UCL Queen Square Institute of Neurology, London, WC1B 5EH, UK; Queen Square MS Centre, Department of Neuroinflammation, UCL Queen Square Institute of Neurology, London, WC1B 5EH, UK; Queen Square MS Centre, Department of Neuroinflammation, UCL Queen Square Institute of Neurology, London, WC1B 5EH, UK; Department of Neurology, Great Ormond Street Hospital for Children, London, WC1N 1LE, UK; Queen Square MS Centre, Department of Neuroinflammation, UCL Queen Square Institute of Neurology, London, WC1B 5EH, UK; Neuroradiology Department, National Hospital for Neurology and Neurosurgery, London, WC1N 3BG, UK; Neuroradiology Department, King's College Hospital NHS Foundation, London, SE5 9RS, UK; Neurology Department, NHS, Redditch, WC1N 3BG, UK; Queen Square MS Centre, Department of Neuroinflammation, UCL Queen Square Institute of Neurology, London, WC1B 5EH, UK; Queen Square MS Centre, Department of Neuroinflammation, UCL Queen Square Institute of Neurology, London, WC1B 5EH, UK; Department of Neurology, Great Ormond Street Hospital for Children, London, WC1N 1LE, UK; Centre for Medical Image Computing, Department of Medical Physics and Biomedical Engineering, University College London, London, WC1V 6LJ, UK; eHealth Center, Universitat Oberta de Catalunya, Barcelona, 08018, Spain; Queen Square MS Centre, Department of Neuroinflammation, UCL Queen Square Institute of Neurology, London, WC1B 5EH, UK; National Institute for Health and Care Research (NIHR) University College London Hospitals (UCLH) Biomedical Research Centre, London, WC1E 6BT, UK; Centre for Medical Image Computing, Department of Medical Physics and Biomedical Engineering, University College London, London, WC1V 6LJ, UK; Department of Radiology and Nuclear Medicine, VU University Medical Centre, Amsterdam, 1081 HV, Netherlands; Queen Square MS Centre, Department of Neuroinflammation, UCL Queen Square Institute of Neurology, London, WC1B 5EH, UK; National Institute for Health and Care Research (NIHR) University College London Hospitals (UCLH) Biomedical Research Centre, London, WC1E 6BT, UK; Neuroradiology Department, National Hospital for Neurology and Neurosurgery, London, WC1N 3BG, UK; Queen Square MS Centre, Department of Neuroinflammation, UCL Queen Square Institute of Neurology, London, WC1B 5EH, UK; National Institute for Health and Care Research (NIHR) University College London Hospitals (UCLH) Biomedical Research Centre, London, WC1E 6BT, UK; Neuroradiology Department, National Hospital for Neurology and Neurosurgery, London, WC1N 3BG, UK

**Keywords:** multiple sclerosis, choroid plexus, pathogenesis, MRI, cognition

## Abstract

Choroid plexus enlargement is a promising biomarker of disease activity in multiple sclerosis. However, longitudinal changes in choroid plexus volume and microstructural integrity remain unclear. This study investigated temporal changes in choroid plexus measures and their correlations with clinical disability and brain volume changes over 18 months and the entire disease duration. We recruited consecutive relapsing-remitting multiple sclerosis patients at treatment initiation who were then invited to come back for clinical, neuropsychological and brain MRI assessments at 6 and 18 months. Choroid plexus volume was measured using FreeSurfer and Gaussian Mixture Models on 3D-T1-weighted scans, and choroid plexus T1/T2 ratio was calculated from conventional 3D-T1- and T2-weighted images. Brain lesion, whole brain, grey matter, and white matter volumes were measured. Alternating conditional expectation algorithm was used to estimate trajectories of changes in choroid plexus measures over the entire disease course. Multiple linear regression and mixed effects models were used to investigate associations of choroid plexus measures with clinical and MRI measures. False discovery rate correction was applied. 422 RRMS patients were recruited [mean age: 40.8 years (SD 10.9), mean disease duration: 9.5 years (SD 17.4), median expanded disability status scale: 2.0 (IQR: 1.5–3.5); mean symbol digit modalities test score: 50.6 (SD 14.7), mean brief visuospatial memory test-revised score: 25 (SD7.6)]; 276 participants were studied at 6-months follow-up and 80 at 18-months. During the entire disease course, an initial increase in normalized choroid plexus volume was observed, followed by a plateau; T1/T2 ratio decreased initially, but then increased once the volume had stabilized. When examining changes in choroid plexus volumes over a median follow-up of 8.6 months, significant increases in both choroid plexus volumes [β = 0.45, standard error = 0.11, False discovery rate-corrected *P* < 0.001)] and T1/T2 ratios (β = 0.29, standard error = 0.14, False discovery rate-corrected *P* = 0.05) were observed. A higher baseline choroid plexus T1/T2 ratio was linked to a faster rate of decrease in normalized brain volume (β = −0.21, standard error = 0.08, False discovery rate-corrected *P* = 0.01) and deep grey matter volume (β = −0.25, standard error = 0.10, False discovery rate-corrected *P* = 0.03) over time. Higher baseline choroid plexus T1/T2 values were associated with worsening performance on brief visuospatial memory test-revised over time (β = −0.23, standard error = 0.10, False discovery rate-corrected *P* = 0.04). Changes in choroid plexus measures over time appear non-linear, with volumes increasing earlier in the disease course and T1/T2 ratios rising later. After a mean disease duration of 9.5 years, higher choroid plexus T1/T2 ratios, but not volume, predicted faster memory decline and whole brain and deep grey matter volume loss, underscoring the value of assessing choroid plexus microstructure, alongside volumes, in predicting clinical and MRI outcomes.

## Teaser

Choroid plexus (ChP) volume increases early in relapsing-remitting multiple sclerosis (RRMS), stabilizing later, while T1/T2 ratio values rise subsequently. Higher baseline T1/T2 ratios predict greater brain atrophy and visuospatial memory decline over 18 months. These findings highlight ChP microstructure alongside with volume as a key biomarker for clinical and MRI outcomes in RRMS.

## Graphical Abstract

**Graphical Abstract:**
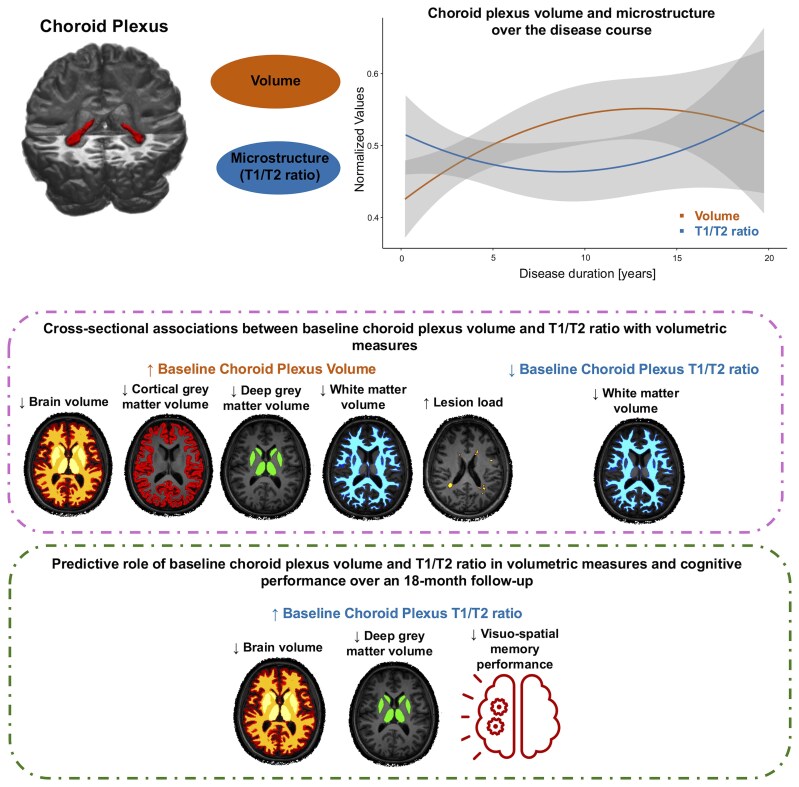

## Introduction

In multiple sclerosis (MS), inflammation occurs not only in the central nervous system (CNS) parenchyma, but also in the surrounding meninges and intrathecal space.^1^ There is growing evidence that inflammation outside the CNS influences pathology within the brain and spinal cord,^1^ and leads to progressive disability^1-4^; this inflammatory activity can become the target of new treatments^5^ and biomarkers of inflammation outside the brain parenchyma can be used to predict treatment response.^6^

Among the extra-parenchymal structures which are affected by inflammation in MS, there is the choroid plexus (ChP) that generates the cerebrospinal fluid that fills the subarachnoid space. The ChP also plays a crucial role in immunosurveillance trough the CCR6-CCL20 axis^7^ and the expression of ICAM-1, VCAM-1, MAdCAM-1 and MHC class I and II molecules that facilitate T cells access to the CNS,^8^ as shown in post mortem studies.^7-9^ Recent studies using magnetic resonance imaging (MRI) have shown enhancement^10^ and enlargement^11^ of ChP in people with MS when compared to healthy subjects. ChP enlargement is associated with: (i) less remyelination in white matter (WM) lesions (quantified by using ^11^C-PiB-PET),^12^ (ii) chronic lesion growth and progressive lesional tissue damage (as measured by diffusion MRI)^13^ and (iii) greater concurrent disability.^12^ The exact mechanisms underlying ChP enlargement are unknown. Studies in experimental autoimmune encephalomyelitis have reported changes in the expression of genes encoding adhesion molecules, interleukins, and T-cell activation markers, which may be responsible for ChP enlargement by promoting the extravasation of autoreactive T cells from the circulation into the ChP stromal compartment.^14^ Degenerative changes (including thickening of basement membrane, fibrosis, and gliosis) may also contribute to ChP enlargement.^15^

To date, only few studies have assessed longitudinal changes in ChP volumes, reporting contrasting results: while some studies observed increase in ChP volume over time,^16^ which was associated with development of brain atrophy and chronic active lesion expansion,^17^ other studies reported ChP volume stability and no association between ChP volume and changes in clinical measures.^18^ No studies have looked yet at the microstructural integrity of the ChP, assessed using the T1/T2 ratio, which is a recently introduced quantitative MRI measure, easily obtainable from conventionally acquired clinical images. When measured in the brain parenchyma, the T1/T2 ratio is sensitive to myelin content, axonal/dendritic structure, and factors such as iron dysregulation, astrogliosis, and inflammation.^19^

In the present study, we assessed ChP volume and T1/T2 ratio at baseline, 6 and 18 months of follow-up, in a prospectively recruited cohort of MS patients. We aimed to investigate: (i) The temporal trajectory of ChP volumes and T1/T2 ratios over both 18-months follow-up and the entire disease duration, using an alternating conditional expectation (ACE) algorithm^20^; (ii) The association between baseline ChP measures and changes in disability (including cognitive performance) and changes in other brain MRI measures over 18 months. To complete the investigation, we also assessed cross-sectional associations between ChP measures, conventional MRI measures, and physical disability and cognitive performance.

## Materials and methods

### Participants

We recruited prospective patients who fulfilled the following inclusion criteria: (i) Diagnosis of relapsing-remitting (RR) MS; (ii) within 3 months from initiation of a disease-modifying treatment (DMT). Participants were invited to undergo clinical, neuropsychological and MRI assessments at three time points: baseline, 6 months and 18 months [the POINT-MS (Predicting Optimal INdividualized Treatment response in MS) cohort].

The study was approved by the local Research Ethics Committee (19/WA/0157) and all participants gave written informed consent at enrolment.

### Neurological assessment

At each visit, patients had a neurological assessment to determine the Expanded Disability Status Scale (EDSS) score.^21^ Detailed information on relapses (date and degree of remission [complete/incomplete clinical recovery within 6 months or no remission]) and DMT use was collected. DMTs were grouped into moderate/low efficacy (interferon-beta, glatiramer acetate, dimethyl fumarate, teriflunomide, fingolimod and cladribrine) and high-efficacy treatments (natalizumab, rituximab, ocrelizumab, ofatumumab and alemtuzumab).^22^

### Neuropsychological evaluation

The Brief International Cognitive Assessment for MS (BICAMS) was used.^23^ The BICAMS assesses the most frequently impaired cognitive domains in MS, incorporating tests of: information processing speed [symbol digit modalities test (SDMT)^24^], learning and memory [California verbal learning test (CVLT-II)^25^ and the brief visuospatial memory test-revised (BVMT-R)^26^].

### MRI acquisitions

A Philips Ingenia CX 3 Tesla scanner (Philips Medical System, Best, The Netherlands) was used to obtain the following at baseline and follow-up scans: 3DT1-gradient echo (GRE, for volumetric measures and segmentation: 1 × 1 × 1 mm, TR = 6.9 ms, TE = 3.1 ms, flip angle = 8°), 2DT2-turbo spin echo (1 × 1 × 3 mm, TR = 3500 ms, TE = 8/19 ms, turbo-factor = 10) to assist lesion identification and 3D fluid-attenuated-inversion recovery (FLAIR, for lesion identification: 1 × 1 × 1 mm, TR = 8000 ms, TE = 388 ms, turbo-factor = 120, inversion time = 2400 ms). For all sequences, slices were positioned to run parallel to a line joining the most infero-anterior and infero-posterior margins of the corpus callosum.

### MRI data analysis

Hyperintense lesions were identified on FLAIR images using a convolutional neural network-based method.^27^ This segmentation was visually reviewed by an experienced observer (EDM). Normalized brain volume, cortical grey matter volume, deep grey matter volume, white matter volume and lateral ventricle volume were measured on the 3DT1-weighted scans using the FreeSurfer software version 7.4.1 (https://surfer.nmr.mgh.harvard.edu/fswiki/FreeSurferWiki) after T1-hypointense lesion filling.^28^ The FreeSurfer longitudinal pipeline (https://surfer.nmr.mgh.harvard.edu/fswiki/FsTutorial/LongitudinalTutorial) was applied to serial scans, and segmentation again reviewed (by EDM). Normalized volumes were obtained by dividing each tissue volume by the total intracranial volume.

### ChP segmentation

Starting from FreeSurfer segmentation, to refine ChP segmentation, we applied Gaussian Mixture Models (GMM) as previously used by Tadayon *et al*. (https://github.com/EhsanTadayon/choroid-plexus-segmentation).^29^ Briefly, a Bayesian GMM with two components was applied to all voxels within the lateral ventricle mask obtained from FreeSurfer segmentation, to group these into two clusters according to their intensity. The higher intensity cluster of voxels is then smoothed by using 3D Susan smoothing algorithm implemented in FSL software (sigma = 1 mm).^30^ A second Bayesian GMM with three components is applied to select the voxels with highest average voxel intensity, then considered as the final ChP.

**Figure 1:**
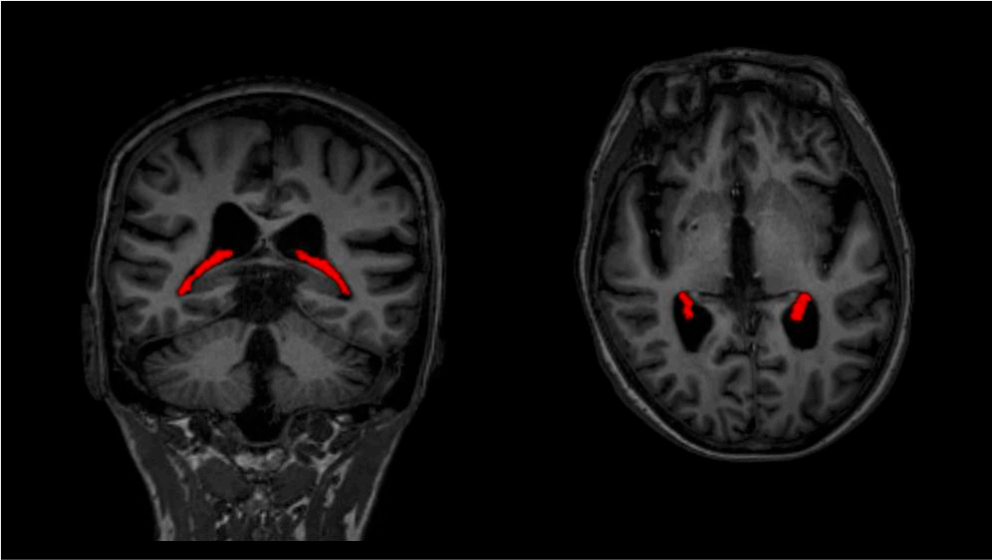
**Choroid plexus.** Example of choroid plexus as segmented on 3DT1-weighted images.

ChP segmentation was reviewed and manually edited when needed by an experienced observer (EDM). An example of ChP is shown in Fig. 1. To obtain a normalized measure for ChP (NChP) volume we divided the ChP volume by the total intracranial volume.^31^

### T1/T2 ratio maps

3D T1-weighted and T2-weighted images were pre-processed and combined using a dedicated pipeline adapted from Ganzetti *et al*.,^32^ implemented in the MATLAB® environment. T2-weighted images were first co-registered to the 3D T1-weighted image space through a rigid-body transformation using FLIRT tool (FSL Library) and then both sequences underwent the intensity N4 bias field correction from the ANTs toolbox (http://stnava.github.io/ANTs/).^33-35^ The 3D T1-weighted and co-registered T2-weighted image intensities were then normalized using a non-linear scaling procedure relative to intensity peaks derived from CSF, bone and soft tissue masks^36,37^ and the ratio between T1 and T2 intensities calculated. The ChP mask was eroded by one voxel (to reduce the potential for partial volume effects) and corresponding T1/T2 ratios extracted.

### Statistical analysis

Demographic, clinical, neuropsychological and MRI features of the study cohort are presented as descriptive statistics, using either mean and standard deviation (SD), frequencies or median and interquartile ranges according to the variable distribution and category. Normality was assessed by the Shapiro–Wilk test and visually inspected by using Q-Q plots. Since no significant difference was observed between left and right NChP, we used the sum of these. To calculate annualized changes, we used the change observed during each available interval. When multiple follow-up intervals were available, the mean of the annualized changes for each interval was calculated. To assess NChP volume and T1/T2 ratio changes over the follow-up time we used mixed effect models, while to characterize the temporal dynamics of ChP and lateral ventricle volume and T1/T2 ratio throughout the disease course, we initially transformed the raw outcome measures into normalized values ranging from 0 to 1, using the empirical cumulative distribution function. We chose this approach to ensure that the variables are considered on a common scale, as done in previous studies applying the same analysis.^20^ Subsequently, ACE algorithm, as described by Donohue *et al*. (https://bitbucket.org/mdonohue/grace/src/master),^20^ was used to estimate long-term trajectories of ChP measures and lateral ventricle volume changes over the disease course from short-term longitudinal observations. To explore associations between NChP volume and microstructure with clinical, neuropsychological measures, and other MRI variables—as well as their temporal changes—we used multiple linear regression and mixed-effects models. Age, gender, disease duration, DMTs (low/moderate and high efficacy groups) and baseline ChP metrics (where relevant) were used as covariates along with random intercepts for patients. For the multivariable analyses, a stepwise variable selection procedure was used. Additionally, noting the association between ChP and lateral ventricle volumes,^38-40^ we re-ran analyses to explore NChP volume changes over time and their association with clinical, neuropsychological, and MRI variables, including normalized lateral ventricle volumes as a covariate. Except for the multivariable stepwise regression analysis, statistical significance was corrected for multiple comparisons using the Benjamini–Hochberg method, with the threshold for significance set at a corrected *P* < 0.05. Stepwise regression was excluded from this correction as it is a model selection technique, where *P*-values are dynamically influenced by the selection process and are not independent comparisons. Results that remain significant after multiple comparison correction are flagged with *. Statistical analysis was performed using R version 4.2.2.

## Results

The Graphical Abstract highlights the key findings of the study.

### Demographic, clinical and neuropsychological features

Of the 422 people included in this study, 276 (65%) had at least one follow-up [median time: 7.2 months, interquartile range (IQR): 6.0, 9.6 months] and 80 (19%) had two follow-ups (median time: 19.2 months; IQR = 18.0, 22.8 months) with an overall median follow-up of 8.6 months (IQR = 6.6, 16.4 months) (Table 1).

**Table 1:** Summarizes baseline demographic, clinical and neuropsychological features and their annualized changes, in the whole cohort and in patients with and without available follow-up data

	All MS patients	MS patients without follow-up	MS patients with follow-up	*P-*values
Self-identified gender [male/female/not-binary]	150/270/2	46/99/1	104/171/1	0.42
Mean age (SD) [years]	40.8 (10.9)	40.5 (10.6)	41.4 (11.5)	0.47
Mean disease duration (SD) [years]	9.5 (17.4)	8.9 (15.7)	10.7 (20.0)	0.31
DMTs (high efficacy/moderate and low efficacy)	257/165	75/71	182/94	0.19
Median EDSS (IQR)	2.0 (1.5–3.5)	2.0 (1.5–3.0)	2.0 (1.5–3.5)	0.67
Median EDSS annualized changes (IQR)			0.3 (−0.5, 0.8)	0.04^#^
Mean symbol digit modalities test (SDMT) score (SD)	50.6 (14.7)	51.6 (14.0)	48.6 (15.7)	0.07
Mean SDMT annualized changes (SD)			5.7 (11.8)	0.17^#^
Mean brief visuospatial memory test-revised (BVMT-R) score (SD)	25.0 (7.6)	25.6 (7.51)	24.9 (7.54)	0.36
Mean BVMT annualized changes (SD)			1.6 (8.0)	0.009^#,*^
Mean California verbal learning test second edition (CVLT-II) score (SD)	53.1 (12.0)	54.0 (11.4)	52.5 (12.9)	0.22
Mean CVLT-II annualized changes (SD)			4.4 (9.9)	<0.001^#,*^
Mean lesion volume (SD) [ml]	11.9 (14.1)	11.4 (14.6)	12.5 (15.1)	0.59
Mean lesion volume annualized changes (SD) [ml]			1.4 (14.2)	0.10^#^
Mean ^a^normalized brain volume (NBV)×1000 (SD)	774.7 (24.3)	776.7 (25.0)	772.7 (23.0)	0.11
Mean ^a^NBV × 1000 annualized changes (SD)			−0.9 (17.2)	0.001^#,*^
Mean ^a^normalized cortical grey matter volume (NCGMV)×1000 (SD)	416.9 (12.5)	417.9 (12.0)	415.9 (13.1)	0.12
Mean ^a^NCGMV × 1000 annualized changes (SD)			−0.8 (6.6)	0.01^#,*^
Mean ^a^normalized deep grey matter volume (NDGMV)×1000 (SD)	27.5 (3.0)	27.7 (3.0)	27.3 (2.8)	0.32
Mean ^a^NDGMV × 1000 annualized changes (SD)			−0.4 (2.1)	0.01^#,*^
Mean ^a^normalized white matter volume (NWMV)×1000 (SD)	315.7 (16.2)	315.8 (19.7)	311.4 (26.6)	0.06
Mean ^a^NWMV × 1000 annualized changes (SD)			−2.2 (19.0)	0.03^#,*^
Mean ^a^normalized ChP volume (ChPV)×1000 (SD)	1.6 (0.6)	1.6 (0.6)	1.6 (0.6)	0.51
Mean ^a^NChPV × 1000 annualized changes (SD)			0.2 (0.5)	<0.001^#,*^
Mean ChP (ChP) T1/T2 (SD)	0.5 (0.1)	0.5 (0.1)	0.5 (0.1)	0.53
Mean ChP T1/T2 annualized changes (SD)			0.0 (0.0)	0.03^#,*^

SD, standard deviation; IQR, interquartile range; DMTs, disease modifying treatments.

^a^Normalized volumes are obtained dividing the volume of the regions divided by the total intracranial volume.

^*^ *P-*values remaining significant after controlling for multiple comparisons.

^*#*^ *P-*values for time effect in linear mixed effect models for longitudinal data.

### Baseline observations

Demographic, clinical, neuropsychological and MRI measures at the study entry and their annualized changes are reported in Table 1.

#### Associations between choroid plexus volume and microstructure with MRI measures

Associations between NChP volume and ChP T1/T2 ratio values with MRI measures are shown in Table 2. A higher NChP volume was associated with a higher brain lesion load (β = 0.35, 95% confidence intervals (CI) = 0.28, 0.43, *P < 0.001) and lower volumes of cortical (β = −0.18, 95%CI = −0.25, −0.11, *P < 0.001) and deep grey matter (β = −0.29, 95%CI = −0.36, −0.21, *P < 0.001) and white matter (β = −0.22, 95%CI = −0.31, −0.14, *P < 0.001). A higher ChP T1/T2 ratio was significantly associated with greater white matter volumes (β = 0.09, 95%CI = 0.01, 0.18, *P = 0.03). After accounting for normalized lateral ventricle volume, none of the associations between NChP volume and other MRI measures remained statistically significant (Supplementary Table 1).

**Table 2:** Summarizes the baseline associations between normalized ChP volume and T1/T2 ratio with the MRI measures assessed

	Normalized ChP volume (NChPV)*			ChP T1/T2 ratio		
	Std β coefficient (95% CI)	Adjusted *R*^2^	*P-*values	Std β coefficient (95% CI)	Adjusted *R*^2^	*P-*values
Normalized brain volume (NBV)^a^	−0.30 (−0.37, −0.22)	0.08	<0.001*	0.08 (−0.01, 0.16)	0.01	0.07
Normalized cortical grey matter volume (NCGMV)^a^	−0.18 (−0.25, −0.11)	0.07	<0.001*	0.00 (−0.07, 0.08)	0.00	0.93
Normalized deep grey matter volume (NDGMV)^a^	−0.29 (−0.36, −0.21)	0.09	<0.001*	0.07 (−0.01, 0.15)	0.01	0.09
Normalized white matter volume (NWMV)^a^	−0.22 (−0.31, −0.14)	0.09	<0.001*	0.09 (0.01, 0.18)	0.03	0.03*
Lesion load	0.35 (0.28, 0.43)	0.09	<0.001*	0.06 (−0.02, 0.14)	0.02	0.11

Std, standardized; CI, confidence intervals.

^a^Normalized volumes are obtained dividing the volume of the regions divided by the total intracranial volume.

^*^ *P-*values remaining significant after controlling for multiple comparisons.

All analyses adjusted for age, gender, disease duration at assessment and current DMT class.

#### Associations between choroid plexus volume and microstructure with demographic, clinical and neuropsychological measures

Among demographic features, only age was significantly associated with NChP volume (β = 0.16, 95% CI = 0.05, 0.27, *P = 0.009). No other significant associations were observed between demographic variables and either ChP volume or the T1/T2 ratio. Associations between MRI variables and EDSS and subtests of the BICAMS are shown in Table 3. A higher NChP volume was correlated with higher EDSS [β = 0.13, 95%CI = 0.02, 0.24, *P* = 0.02] and lower BVMT-R scores (β = −0.16, 95%CI = −0.27, −0.05, *P* = 0.004), whereas the T1/T2 ratio showed no correlation with clinical and neuropsychological measures (Table 3). These findings were materially unchanged by the addition of normalized lateral ventricle volume to the statistical models.

**Table 3:** Summarizes the results of multivariable stepwise regression analysis between clinical and neuropsychological outcomes with MRI measures

	MRI variables	Std β coefficient (95%CI)	*P-*values	Adjusted *R*^2^
Expanded disability status scale (EDSS)	Normalized choroid plexus volume (NChPV)^a^	0.13 (0.02, 0.24)	0.02	0.24
Symbol digit modalities test (SDMT)	Lesion load	−0.33 (−0.44, −0.21)	<0.001	0.25
	Normalized brain volume (NBV)^a^	0.16 (0.04, 0.27)	0.01	
Brief visuospatial memory test-revised (BVMT-R)	NChPV^a^	−0.16 (−0.27, −0.05)	0.004	0.32
	Lesion load	−0.28 (−0.42, −0.14)	<0.001	
	NBV^a^	0.14 (0.03, 0.25)	0.01	
California verbal learning test second edition (CVLT-II)	Lesion load	−0.19 (−0.33, −0.05)	0.01	0.28
	NBV^a^	0.15 (0.04, 0.26)	0.01	

Std, standardized; CI, confidence intervals.

^a^Normalized volumes are obtained dividing the volume of the regions divided by the total intracranial volume.

All analyses adjusted for age, gender, disease duration at assessment and current DMT class.

### Longitudinal changes

#### Choroid plexus measures over the follow-up and the entire disease duration

Over the follow-up period, NChP showed an overall increase in volume (β = 0.45, 95%CI = 0.23, 0.67, *P < 0.001), even when accounting for normalized lateral ventricle volume as covariate (β = 0.08, 95%CI = 0.02, 0.14, *P = 0.01). Similarly, ChP T1/T2 ratio values increased over the follow-up (β = 0.29, 95%CI = 0.02, 0.56, *P = 0.03).

**Figure 2:**
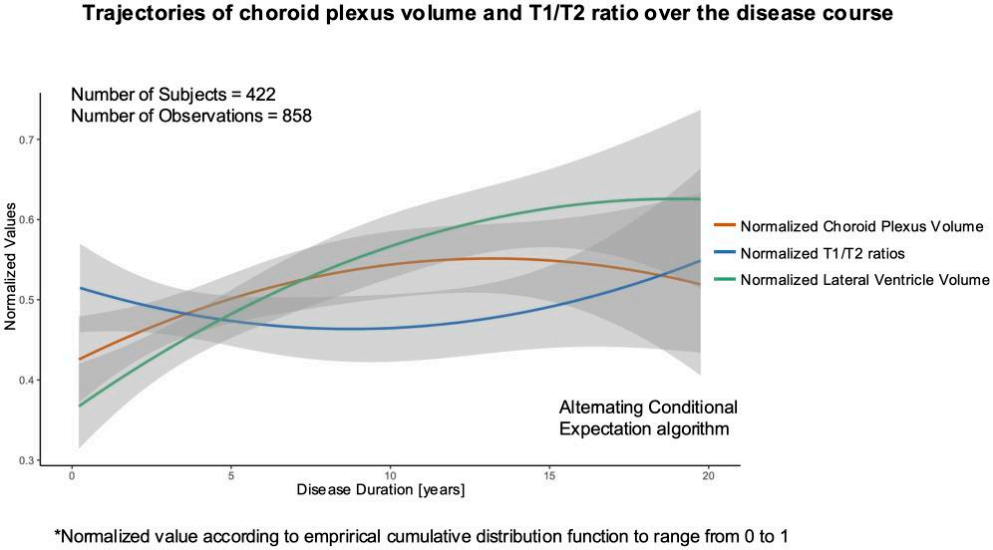
**Trajectories of ChP volume and T1/T2 ratio over the disease course.** The figure shows the trajectories (with 95% confidence intervals, shaded in grey) of ChP volume and T1/T2 ratio values and of lateral ventricle volume over disease duration. All three variables were rescaled using the empirical cumulative distribution function to range from 0 to 1. Alternating conditional expectation (ACE), a non-parametric regression technique, was applied to data from all available time points (422 patients; 858 observations) to estimate the non-linear long-term trajectories.

However, when examining the temporal trajectories of ChP and lateral ventricle measures in relation to disease duration, an initial increase in normalized ChP volume was observed during the first 12 years following disease onset, after which a plateau occurred, followed by a subtle decrease, while the normalized lateral ventricle volume continued to increase. Simultaneously, the T1/T2 ratio initially decreased until 10 years into the disease, but then increased once the ChP volume had stabilized (Fig. 2).

#### MRI measures

There was a significant decrease in normalized brain volume (β = −0.05, 95%CI = −0.07, −0.13, *P = 0.001), cortical (β = −0.03, 95%CI = −0.05, −0.01, *P = 0.01) and deep (β = −0.05, 95%CI = −0.09, −0.01, *P = 0.01) grey matter volumes, and white matter volume (β = −0.04, 95%CI = −0.06, −0.02, *P* = 0.03). Table 4 reports the associations between MRI measures and follow-up time.

**Table 4:** Summarizes the time, and the time × baseline normalized ChP volume and T1/T2 ratio value interaction effect on the changes of the remaining MRI measures obtained

	Time		Time × baseline normalized ChP volume		Time × baseline ChP T1/T2 ratio	
MRI variables	Std β coefficient (95% CI)	*P-*values	Std β coefficient (95% CI)	*P-*values	Std β coefficient (95% CI)	*P*-values
Normalized brain volume (NBV)^a^	−0.05 (−0.07, −0.03)	0.001*	0.07 (−0.03, 0.16)	0.21	−0.21 (−0.37, −0.05)	0.005*
Normalized cortical grey matter volume (NCGMV)^a^	−0.03 (−0.05, −0.01)	0.01*	0.04 (−0.06, 0.14)	0.44	−0.13 (−0.27, 0.00)	0.05
Normalized deep grey matter volume (NDGMV)^a^	−0.05 (−0.09, −0.01)	0.01*	0.09 (−0.05, 0.23)	0.21	−0.25 (−0.45, −0.05)	0.01*
Normalized white matter volume (NWMV)^a^	−0.04 (−0.06, −0.02)	0.03	0.06 (−0.04, 0.16)	0.39	−0.16 (−0.30, −0.02)	0.03
Lesion load	−0.03 (−0.11, 0.05)	0.10	0.02 (−0.01, 0.14)	0.64	0.10 (−0.06, 0.26)	0.19

Std, standardized; CI, confidence intervals.

^a^Normalized volumes are obtained dividing the volume of the regions divided by the total intracranial volume.

^*^ *P*-values remaining significant after controlling for multiple comparisons.

All analyses adjusted for age, gender, disease duration at assessment, and current DMT class.

#### Clinical and neuropsychological measures

We observed a significant increase in EDSS scores over the follow-up period (β = 0.03, 95%CI = 0.03, 0.09, *P* = 0.04), alongside improved performance on the BVMT-R (β = 0.05, 95%CI = 0.01, 0.09, *P* = *0.009) and CVLT-R (β = 0.13, 95%CI = 0.09, 0.17, *P < 0.001). Table 5 reports the all the associations between clinical and neuropsychological measures and follow-up time.

**Table 5:** Summarizes the time and the time × baseline MRI variable interaction effect on the EDSS and neuropsychological measure changes over time

	MRI variables	Expanded disability status scale (EDSS)		Symbol digit modalities test (SDMT)		Brief visuospatial memory test-revised (BVMT-R)		California verbal learning test second edition (CVLT-II)	
		Std β coefficient (95% CI)	*P-*values	Std β coefficient (95% CI)	*P-*values	Std β coefficient (95% CI)	*P-*values	Std β coefficient (95% CI)	*P-*values
Time		0.03 (0.01, 0.50)	0.04	0.03 (−0.01, 0.07)	0.17	0.05 (0.01, 0.09)	0.009*	0.13 (0.09, 0.17)	<0.001*
Interaction Time x	Normalized choroid plexus volume (NChPV)^a^	0.01 (−0.11, 0.13)	0.85	0.00 (−0.12, 0.12)	0.99	0.18 (−0.02, 0.38)	0.08	0.06 (−0.08, 0.20)	0.41
	ChP T1/T2 ratio	0.08 (−0.10, 0.26)	0.39	−0.03 (−0.21, 0.15)	0.69	−0.23 (−0.43, −0.03)	0.02*^,#^	0.11 (−0.09, 0.31)	0.27
	Normalized brain volume (NBV)^a^	0.30 (−1.03, 1.63)	0.32	0.33 (−1.18, 1.84)	0.36	−1.48 (−3.28, 0.32)	0.11	−1.39 (−3.06, 0.28)	0.10
	Normalized cortical grey matter volume (NCGMV)^a^	0.10 (−1.17, 1.37)	0.88	1.86 (0.49, 3.23)	0.01	−1.40 (−3.11, 0.31)	0.11	2.20 (0.18, 4.22)	0.03
	Normalized deep grey matter volume (NDGMV)^a^	−0.12 (−0.47, 0.23)	0.48	0.04 (−0.06, 0.14)	0.44	0.35 (−0.08, 0.78)	0.11	−0.18 (−0.57, 0.21)	0.37
	Normalized white matter volume (NWMV)^a^	0.32 (−0.46, 1.10)	0.43	−0.66 (−1.89, 0.56)	0.11	−0.79 (−1.79, 0.21)	0.12	−1.16 (−2.67, 0.35)	0.11
	Lesion load	0.07 (0.01, 0.13)	0.01^§^	−0.02 (−0.08, 0.04)	0.53	−0.05 (−0.13, 0.03)	0.13	−0.01 (−0.07, 0.05)	0.77

Std, standardized; CI, confidence intervals.

^a^Normalized volumes are obtained dividing the volume of the regions divided by the total intracranial volume.

^*^ *P-*values remaining significant after controlling for multiple comparisons.

^#^Surviving at stepwise regression analysis.

All analyses adjusted for age, gender, disease duration at assessment and current DMT class.

### Baseline predictors of longitudinal changes

#### Associations between baseline NChP volume and ChP T1/T2 with changes of MRI measures over the follow-up time

Associations between baseline NChP volume and ChP T1/T2 with MRI variable changes over time are reported in Table 4, while those accounting for normalized lateral ventricle volume are reported in Supplementary Table 2.

A higher baseline ChP T1/T2 ratio was linked to a faster rate of normalized brain (β = −0.21, 95%CI = −0.36, −0.05, *P = 0.005), cortical (β = −0.13, 95%CI = −0.25, −0.01, *P* = 0.05) and deep (β = −0.25, 95%CI = −0.45, −0.05, *P = 0.01) grey matter and white matter (β = −0.16, 95%CI = −0.30, −0.02, *P* = 0.03) volume loss over time. Conversely, baseline NChP volume was not associated with changes in MRI measures over time, and this did not change when accounting for normalized lateral ventricle volume.

#### Associations between baseline NChP volume and ChP T1/T2 with changes of clinical and neuropsychological measures over the follow-up time

Associations between baseline NChP volume and ChP T1/T2 with MRI variable changes over time are reported in Table 5, while those accounting for normalized lateral ventricle volume are reported in Supplementary Table 3. Higher baseline ChP T1/T2 values were associated with worsening BVMT-R performance over time (β = −0.23, 95%CI = −0.43, −0.03, *P = 0.02). Baseline NChP volume was not associated with significant changes in clinical and neuropsychological measures, and this did not change when accounting for normalized lateral ventricle volume.

## Discussion

We found that after a mean disease duration of 9.5 years, both the volume and structural integrity of the ChP changed over time, but not in parallel. While both ChP volumes and T1/T2 ratios increased over a median follow-up of 8.6 months, their trajectories differed when mapped against the entire disease duration. ChP volumes initially increased, plateaued around 12 years of disease duration, and showed a subtle decline thereafter. In contrast, T1/T2 ratios initially decreased reaching a nadir at about 8 years of disease duration, before beginning to rise. Associations with subsequent clinical outcomes were observed principally with ChP T1/T2 ratios rather than volumes. This suggests that volume alone does not fully reflect clinically relevant ChP pathology in MS, and that a measure of tissue integrity provides complementary insights.

Several mechanisms may contribute to the enlargement of the ChP, including the overexpression of genes associated with oxidative stress response and its consequent mitochondrial dysfunction, as well as increased plasma leakage across the blood-cerebrospinal fluid (CSF) barrier.^41,42^ An association has been reported between larger ChP volume and longer pseudo-T2 relaxation times,^18^ a putative marker of inflammation, suggesting that inflammation may be an underlying mechanism of enlarged ChP. It is possible that inflammation, which is associated with increase water content, is thus responsible for the initial decrease in T1/T2 ratio. However, leakage of the blood-CSF barrier is not the sole mechanism potentially responsible for ChP enlargement, which has also been linked to endothelial immune proliferation and the migration of immune cells,^8,43^ including the infiltration of non-resident immune cells into the ChP.^44^ Indeed, an association between greater ChP volume and higher translocator protein (TSPO) expression has been reported likely reflecting increased macrophage/microglia infiltration in the ChP.^31^ Activated macrophages and microglia are characterized by increased iron content.^5^ Iron is known to shorten T2 relaxation times, leading to a reduction in signal intensity on T2-weighted images.^45^ In line with this, animal models of neuroinflammation have shown elevated levels of iron in the ChP.^46^ This supports the hypothesis that iron accumulation—likely due to macrophage and microglial infiltration^31^—may underlie the delayed increase in T1/T2 ratio values.

Confirming previous studies^38-40^ we found an association between ChP and lateral ventricle volume, and noting that lateral ventricle volume is itself related to brain tissue volumes^47,48^ and correlates with brain WM lesion loads,^49^ it is perhaps unsurprising that including normalized lateral ventricle volumes in our statistical models rendered all associations between NChP volume and other MRI variables non-significant. It emphasizes that these elements of pathology are all interrelated but does not lead us to conclude that ChP volume changes are solely a function of lateral ventricular enlargement. Here, it is also worth noting that the ChP T1/T2 ratio did not simply change in parallel to either ChP or lateral ventricle volumes, and so making it unlikely that they are just the consequence of lateral ventricle expansion. This is also in line with evidence reporting higher ChP volume without lateral ventricle enlargement in the earliest stages of disease. Higher ChP volume has indeed been observed in patients with clinically isolated syndrome compared to healthy subjects,^50^ whereas no differences were found in lateral ventricle volume between these groups.^51^ Further, including lateral ventricle volume in predictive models of neurological disability and cognitive functioning did not materially affect associations with NChP volumes, as also observed in a previous study.^40^ This suggests that ChP pathology has an independent effect on clinical outcomes that is not be mediated by either brain WM lesions or atrophy. It remains unknown how such effects are mediated but, for example, the ChP may play a role in shaping cognitive functioning by modulating the migration of immune cells within the CNS^8,43^ and through changes in the inflammatory CSF profile.^52^

Considering the temporal relationship between ChP volume increases, ChP T1/T2 ratios and lateral ventricle expansion, no ChP measure mirrors lateral ventricle volumes. This suggests time-dependent associations, and different elements of ChP pathology may be associated with ventricular enlargement at different times; however, in the present study we cannot establish a clear causal relationship, i.e. a change in ChP measures preceding ventricular enlargement, or vice versa. The baseline predictive models do however give some indication of a plausible direction of travel, showing the ChP T1/T2 ratios (rather than volumes) predicted a faster rate of normalized brain volume loss.

With the caveat that most associations with clinical outcomes were not significant, consistent with baseline ChP T1/T2 ratios (rather than volumes) predicting brain tissue pathology, they also predicted a faster decline in visuospatial memory (BVMT-R) performance. The mean duration of follow-up was less than a year and there were significant improvements in cognitive scores (BVMT-R and CVLT-R), so it would be of interest to look at these associations again with longer term follow-up, re-baselining cognitive measures to allow for potential early learning or initial anti-inflammatory treatment effects.

This study has limitations. Firstly, we did not include healthy participants in our analysis, which would have enabled us to characterize the extent of abnormalities in volume (which have been previously described^31^) and T1/T2 ratio in patients when compared with healthy controls. Our study exclusively focused on people with relapsing-remitting MS, thus limiting the generalizability of our findings to other disease phenotypes. In addition, all participants initiated a treatment, and early treatment effects may have disrupted some associations (for example through pseudo-atrophy^53^). The relatively short follow-up duration (up to a maximum of 24 months) will have constrained our ability to detect disability progression and, in particular, cognitive decline as this may in part be masked by learning effects.^54^ However, a learning effect can be considered an outcome in itself,^54^ indicating that ChP microstructural changes can negatively influence this.

In conclusion, our findings suggest that both tissue volume and microstructure should be assessed when seeking to characterize pathological processes involving the ChP, and their impact on CNS tissue damage and clinical outcomes.

## Supplementary Material

fcaf239_Supplementary_Data

## Data Availability

The data that support the findings of this study are available from the corresponding author upon reasonable request. The pipeline employed to generate T1/T2 ratio maps is publicly available via the MRTool platform (https://www.nitrc.org/projects/mrtool/). For choroid plexus segmentation, we adapted a publicly available algorithm (https://github.com/EhsanTadayon/choroid-plexus-segmentation). The Alternating Conditional Expectation (ACE) algorithm used for statistical modelling can be accessed at https://bitbucket.org/mdonohue/grace/src/master.
