# Supplementary material for: The temporal dynamics and clinical relevance of choroid plexus measures in multiple sclerosis
Source: Brain Commun. 2025 Jun 14;7(3):fcaf239. doi: 10.1093/braincomms/fcaf239 (PMC12203544; doi:10.1093/braincomms/fcaf239)
Supplement: fcaf239_Supplementary_Data [file fcaf239_supplementary_data.docx]

**Supplementary Materials**

- **Choroid plexus volume and lateral ventricle**
- **Table 1**
- **Table 2**
- **Table 3**

**Choroid plexus volume and lateral ventricle**

We observed significant correlation between NChP volume and normalized lateral ventricle volume at baseline [β=0.53, standard error (SE) =0.05, **p*<0.001] and over follow-up time (β=0.25, SE =0.06, **p*<0.001) but not over the disease duration.

**Table 1.** Summarizes the baseline associations between normalized choroid plexus volume with the MRI measures assessed, accounting for the normalized lateral ventricle volume.

|  | ***Normalized choroid plexus volume (NChPV)** | | |
| --- | --- | --- | --- |
| **MRI variables** | **Std β coefficient**  **(95%CI)** | **Adjusted R^2^** | ***p***  **values** |
| ***Normalised brain volume (NBV)** | -0.03  (-0.16, 0.09) | 0.28 | 0.58 |
| ***Normalized cortical grey matter volume (NCGMV)** | 0.14  (0.03, 0.26) | 0.38 | 0.10 |
| ***Normalized deep grey matter volume (NDGMV)** | -0.05  (-0.18, 0.07) | 0.31 | 0.40 |
| ***Normalized white matter volume (NWMV)** | -0.16  (-0.29, -0.02) | 0.12 | 0.07 |
| **Lesion load** | 0.07  (-0.05, 0.19) | 0.35 | 0.26 |

Abbreviations: Std=standardized; CI=Confidence intervals.

*Normalized volumes are obtained dividing the volume of the regions divided by the total intracranial volume.

All analyses adjusted for age, gender, disease duration at assessment, current DMT class and normalised lateral ventricle volume.

**Table 2.** Summarizes the time x baseline normalized choroid plexus volume interaction effect on the changes of the remaining MRI measures obtained accounting for normalised lateral ventricle volume.

|  | **Time x**  **baseline normalized choroid plexus volume** | |
| --- | --- | --- |
| **MRI variables** | **Std β coefficient**  **(95%CI)** | ***p***  **values** |
| ***Normalised brain volume (NBV)** | -0.01  (-0.11, 0.09) | 0.85 |
| ***Normalized cortical grey matter volume (NCGMV)** | -0.02  (-0.06, 0.02) | 0.29 |
| ***Normalized deep grey matter volume (NDGMV)** | -0.01  (-0.07, 0.05) | 0.91 |
| ***Normalized white matter volume (NWMV)** | -0.03  ( -0.07, 0.01) | 0.38 |
| **Lesion load** | 0.01  (-0.03, 0.05) | 0.44 |

Abbreviations: Std=standardized; CI=Confidence intervals.

*Normalized volumes are obtained dividing the volume of the regions divided by the total intracranial volume.

All analyses adjusted for age, gender, disease duration at assessment, current DMT class and normalised lateral ventricle volume.

**Table 3.** Summarizes the time x baseline normalized choroid plexus volume interaction effect on the EDSS and neuropsychological measure changes over time accounting for normalised lateral ventricle volume.

|  |  | **Expanded disability status scale (EDSS)** | | **Symbol digit modalities test (SDMT)** | | **Brief visuospatial memory test-revised**  **(BVMT-R)** | | **California verbal learning test second edition**  **(CVLT-II)** | |
| --- | --- | --- | --- | --- | --- | --- | --- | --- | --- |
|  |  | **Std β coefficient**  **(95%CI )** | ***p***  **values** | **Std β coefficient**  **(95%CI )** | ***p***  **values** | **Std β coefficient**  **(95%CI )** | ***p***  **values** | **Std β coefficient**  **(95%CI )** | ***p***  **values** |
| **Interaction**  **Time x** | ***Normalized choroid plexus volume (NChPV)** | 0.02  (-0.02, 0.06]) | 0.42 | 0.03  (-0.03, 0.09) | 0.38 | 0.02  (-0.04, 0.08) | 0.55 | -0.02  (-0.08, 0.04) | 0.46 |

Abbreviations: Std=standardized; CI=Confidence intervals.

*Normalized volumes are obtained dividing the volume of the regions divided by the total intracranial volume.

All analyses adjusted for age, gender, disease duration at assessment, current DMT class and normalised lateral ventricle volume.
